# Delayed tension gastrothorax caused necrosis of stomach and re-expansion pulmonary edema: a case report

**DOI:** 10.1186/s40792-022-01454-1

**Published:** 2022-05-19

**Authors:** Yuki Mochida, Ryohei Nishizawa, Koji Ochiai, Yoshitaka Inoue, Yasuhiko Kaita, Yoshihiro Yamaguchi

**Affiliations:** Department of Trauma and Critical Care Medicine, Kyorin University School of Medicine, 6-20-2, Shinkawa, Mitaka-shi, Tokyo, 181-8611 Japan

**Keywords:** Thoracotomy, Pulmonary edema, Insufflation, Ischemia

## Abstract

**Background:**

Traumatic tension gastrothorax is a rare and potentially fatal condition occurring in patients with congenital or acquired diaphragmatic defects. Traumatic tension gastrothorax leads to acute and severe respiratory distress. Delayed tension gastrothorax that develops late during injury can be more severe.

**Case presentation:**

An 84-year-old woman was brought to our facility with cardiac arrest and returned to spontaneous circulation after 2 min of cardiopulmonary resuscitation. Computed tomography showed diaphragmatic injury and tension gastrothorax due to trauma because of a fall episode few days earlier. Emergency thoracotomy and laparotomy was performed, because nasogastric tube insertion failed. There was a partially necrotic stomach in the chest cavity. The stomach was retracted from the thoracic cavity into the abdominal cavity and placed in its proper position. There was a 5 cm tear of the diaphragm. The tear was sutured and closed and then the necrotic area of the stomach was resected. Although the surgery relieved the intrathoracic compression, it resulted in re-expansion pulmonary edema immediately after surgery and hypoxemia. The patient was unable to overcome the hypoxemic state and eventually died.

**Conclusions:**

Delayed tension gastrothorax can lead not only to obstructive shock due to intrathoracic compression but also to more severe organ ischemia and re-expansion pulmonary edema due to insufflation.

## Background

Gastrothorax is a rare condition that can be caused by trauma or congenital anomalies [1, 2]. On developing gastrothorax, cardiac decompression and mediastinal deviation can lead to obstructive shock, which is called tension gastrothorax and can be fatal. The priority is to relieve the compression, for which there are effective methods according to published reports, such as insertion of nasogastric tube, gastrointestinal endoscopy, and percutaneous needle decompression [3–8].

Traumatic tension gastrothorax occurs over a wide period from minutes to years after the trauma [1]. However, it is extremely rare that re-expansion pulmonary edema (RPE) and organ ischemia occur after tension gastrothorax. We report our experience of a case of tension gastrothorax which caused severe RPE.

## Case presentation

**Fig. 1:**
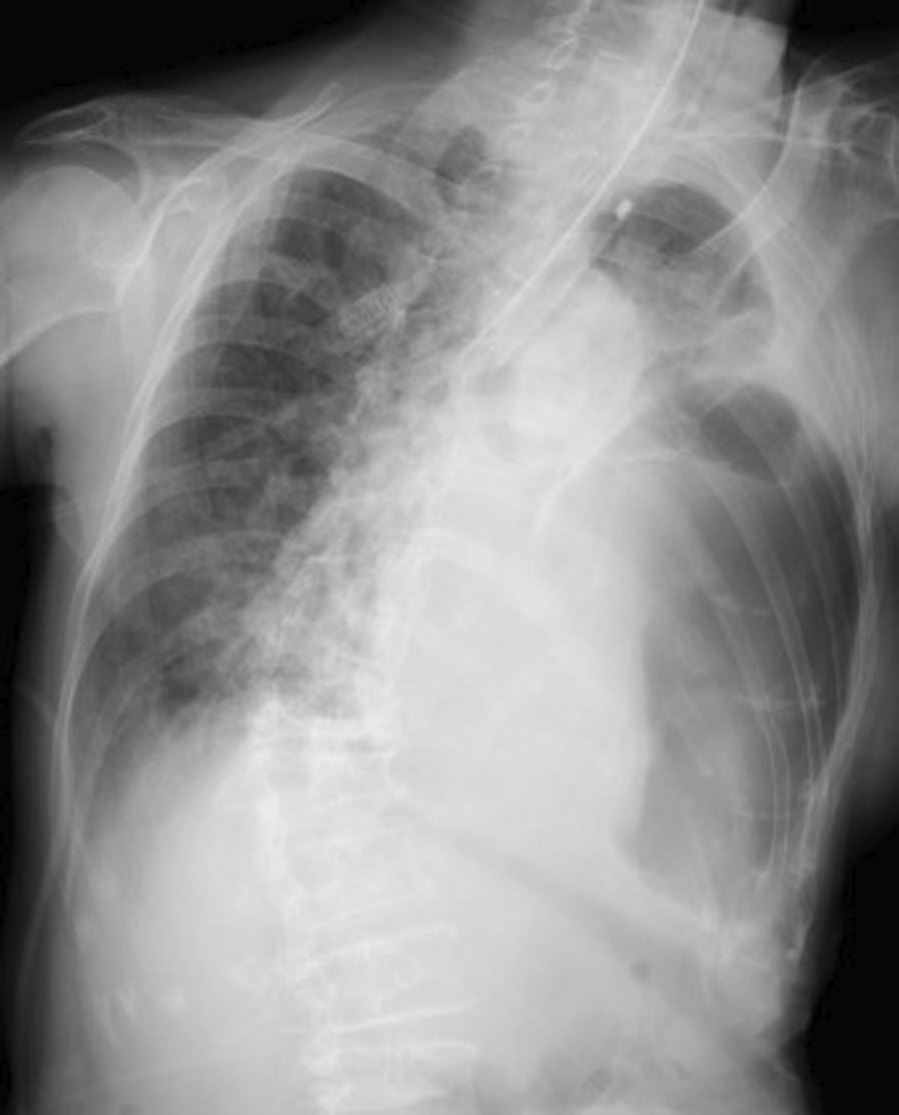
Midline shift of mediastinum. Initial chest radiograph showed an air image in the left middle and lower lung fields

An 84-year-old woman with a history of osteoporosis was brought to our facility because of decreased oxygen saturation (approximately 70%) after vomiting during a meal. The patient stopped breathing and her pulse was not palpable on admission, and chest compressions and adrenaline administration were performed as cardiopulmonary resuscitation. Oral tracheal intubation was performed, and the patient returned to spontaneous circulation after 2 min of chest compressions. A chest radiograph showed an air image from the left middle to lower lung fields (Fig. 1). Chest radiography also showed rib fractures, and the patient was suspected to have diaphragmatic injury and tension gastrothorax due to trauma because of a fall episode that occurred a few days earlier. We attempted to insert a nasogastric tube for decompression; however, this was difficult. Since hemodynamics were maintained by administration of a vasopressor, computed tomography (CT) was performed to confirm the diagnosis.

**Fig. 2:**
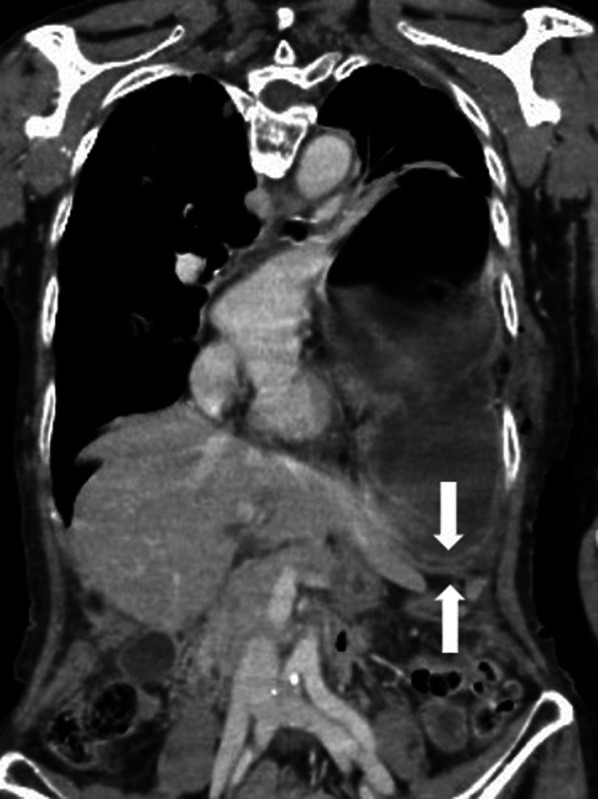
Diagnosis of tension gastrothorax. Computed tomography showed prolapse of the stomach into the thoracic cavity and compression of the left thoracic cavity. The two-layered structure of the gastric wall and diaphragm indicated gastrothorax (arrow)

CT images showed that the stomach was dilated in the thoracic cavity and occupied the left thoracic cavity, compressing the left lung and heart, leading to the diagnosis of tension gastrothorax (Fig. 2). We judged that there was no time for decompression measures other than a nasogastric tube and decided to perform emergency surgery immediately.

Emergency surgery was performed in the right hemi-lateral position to retract the stomach back into the abdominal cavity and to observe the thoracic cavity and repair the diaphragm. A skin incision was placed with an anterolateral thoracotomy, and a paraspinal rectus muscle incision was added to the left ninth intercostal space as a guide.

**Fig. 3:**
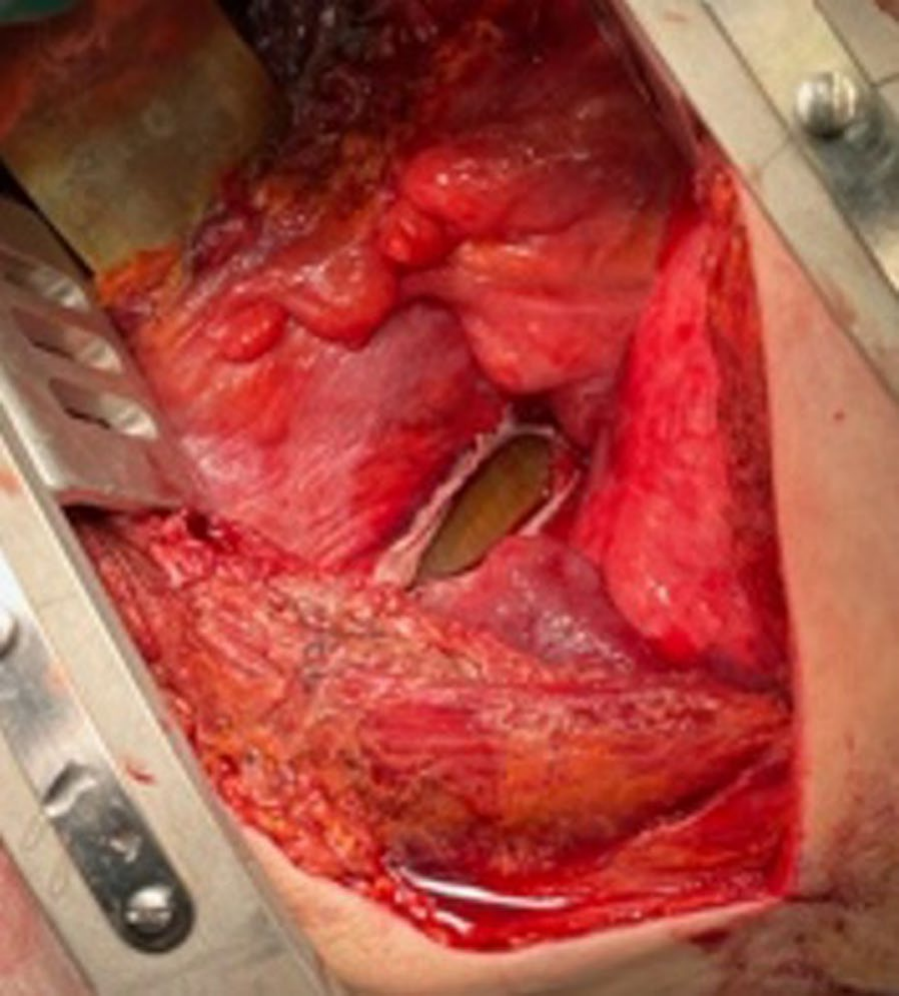
Rupture of diaphragm. A 5 cm laceration was found in the middle of the diaphragm

A distended stomach was observed on opening the chest cavity. The entire stomach was in the thoracic cavity, and extensive black necrosis was observed from the gastric fundus to the mid-gastric region. The lower part of the stomach near the diaphragmatic rupture was not necrotic, but a 1 cm perforation was observed on the lateral side of the greater curvature, through which the gastric contents exuded. A suction tube was inserted into the perforation spot to drain the stomach, and the pressure on the heart was released. Cardiac output improved, blood pressure increased, and hemodynamics stabilized after the pressure was released. The area of the stomach around the perforation site was not necrotic, and it was considered that the fractured rib fragment had punctured and perforated the stomach based on its position in the chest cavity. The stomach was retracted from the thoracic cavity into the abdominal cavity and placed in the abdominal cavity. The diaphragm showed an approximately 5 cm tear, which was sutured and closed (Fig. 3).

The stomach was retracted into the abdominal cavity and observed to have extensive necrosis and thinning of the wall on the greater curvature side from the gastric fundus to the mid-gastric region. The necrotic area was resected horizontally along the line from the hilum to the pylorus, and handsewn anastomosis by Albert–Lembert suture was performed. The operation time was 248 min, and the intraoperative water balance was + 2790 ml (containing 1240 ml of blood transfusion).

**Fig. 4:**
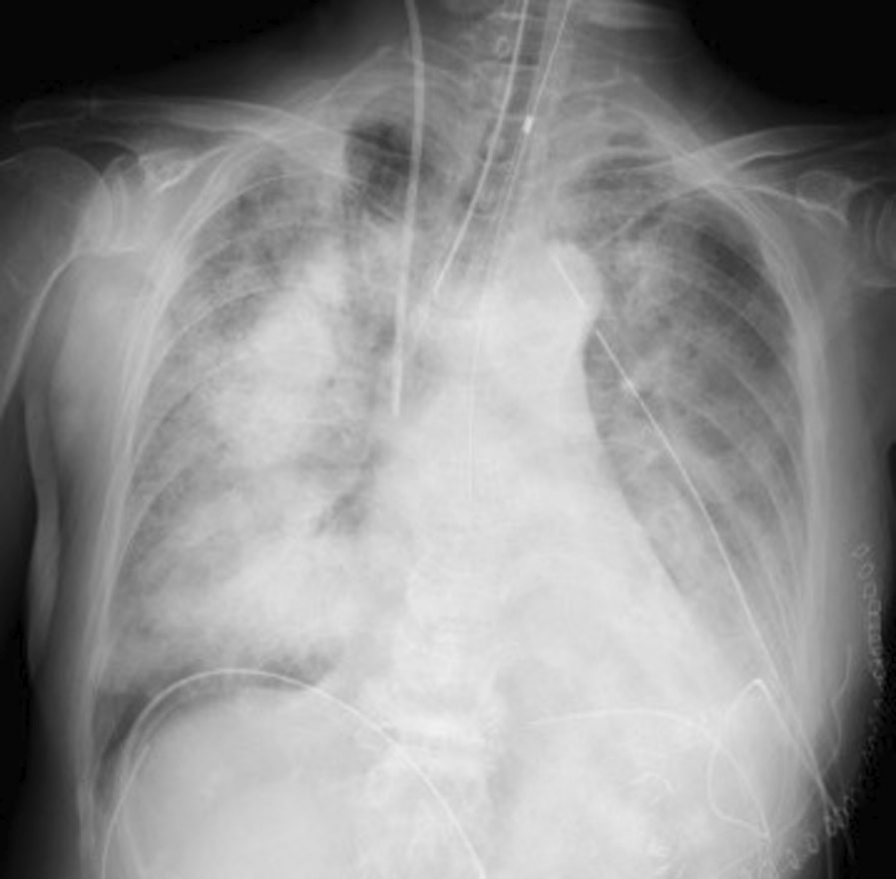
Re-expansion pulmonary edema. Postoperative chest radiograph showed re-expansion pulmonary edema

Although the surgery relieved intrathoracic compression, oxygenation gradually worsened after decompression during surgery, resulting in RPE immediately after surgery, as well as fatal hypoxemia (oxygen partial pressure was 46.7 mmHg under pure oxygen administration) (Fig. 4). Thereafter, ventilator management with pure oxygen was performed for hypoxia; however, hypoxia did not improve. The patient was unable to overcome the hypoxemic state, and hemodynamics were unstable, even with use of a vasopressor. The patient died 8 h after surgery.

## Discussion

The concept of tension gastrothorax was first described in 1984 [1]. Tension gastrothorax, which is caused by traumatic rupture of the diaphragm, is a rare condition with high mortality rates [7]. Traumatic rupture of the diaphragm occurs in approximately 5% of chest or abdominal trauma cases, and gastrothorax occurs in 0.8–8% of diaphragm rupture cases [5, 7]. Prolapse of the stomach into the thoracic cavity leads to gastric dilatation due to impaired gastric emptying, resulting in obstructive shock. Prompt decompression of the stomach is the key to preventing obstructive shock from leading to cardiac arrest [1, 3–7].

The insertion of a nasogastric tube is a simple and rapid method of decompression and is the first choice [1, 3–5, 8]. The success rate of this method is low due to kinking of the stomach at the level of the diaphragm [3, 4]. The next method is percutaneous transthoracic drainage with a needle or chest drain tube or decompression with an endoscope, but the choice of method depends on the facility and the case [3, 4, 6].

Surgical decompression is the final treatment option when these techniques are ineffective. Surgical options include laparotomy and thoracotomy. Thoracotomy is superior in thoracic decompression [3, 4, 7].

In our case, we attempted to insert a gastric tube, but it was unsuccessful; therefore, we chose surgery as a reliable method of decompression. First, the surgery was performed by thoracotomy for rapid decompression of the thoracic cavity, followed by laparotomy to retract the stomach from the thoracic cavity into the abdominal cavity.

The fall trauma that caused the diaphragmatic rupture occurred several days earlier, and the time lapse may have caused organ ischemia. Subsequently, increased abdominal pressure due to vomiting at onset may have caused worsening gastric prolapse and caused delayed-onset traumatic tension gastrothorax. In delayed cases, as in this case, organ necrosis may have occurred, and gastrointestinal surgery (resection and suturing) may be required after retracting the stomach back into the abdominal cavity. Therefore, the patient should be prepared for laparotomy and thoracotomy simultaneously. In this case, the right hemi-lateral position was effective in securing the view of both the thoracic and abdominal cavities. Although surgical decompression of the thoracic cavity and resection and suture reconstruction of the stomach were successful, RPE by decompression of the left lung led to the fatal outcome.

Bilateral RPE is a rare and life-threatening complication in the treatment of lung diseases, such as pneumothorax [9–11]. The exact mechanism of RPE has not yet been completely elucidated [9]. It is associated with a high risk of prolonged lung collapse and a high degree of collapsibility. Although slow drainage is a method to avoid RPE, it is difficult to perform this in the case of tension gastrothorax [10]. RPE is difficult to differentiate from heart failure. The patient did not have history or signs of heart failure. To prevent RPE, differential lung ventilation and slow inflation of the left lung should be considered [12]. In the present case, it was difficult to prepare a differential lung ventilation tube before emergent surgery; therefore, differential lung ventilation should be prepared at the time of admission. Extracorporeal membrane oxygenation was also considered to save the patient's life, but the patient's family refused to undergo the treatment in consideration of her age.

In conclusion, delayed-onset traumatic tension gastrothorax may lead to serious complications, such as organ necrosis, RPE, and obstructive shock. In addition, along with the release of the gastrothorax, open thoracotomy may be effective when additional procedures such as diaphragmatic repair and gastrectomy are considered.

## Conclusions

Although tension gastrothorax is difficult to identify because of its rarity, prompt decompression is critical to prognosis; therefore, sharing information about cases is important. In addition, when tension gastrothorax occurs late, organ ischemia and RPE must be considered, which increases the severity of the disease.

## Data Availability

All data generated or analyzed during this study are included in the manuscript.

## Abbreviations

RPE: Re-expansion pulmonary edema
CT: Computed tomography
